# Relation between body composition and bone mineral density in young undregraduate students with different nutritional status

**DOI:** 10.1590/S1679-45082016AO3569

**Published:** 2016

**Authors:** Edil de Albuquerque Rodrigues, Marcos André Moura dos Santos, Amanda Tabosa Pereira da Silva, Breno Quintella Farah, Manoel da Cunha Costa, Florisbela de Arruda Camara e Siqueira Campos, Ana Patrícia Siqueira Tavares Falcão

**Affiliations:** 1 Universidade de Pernambuco, Recife, PE, Brazil.; 2 Universidade Federal de Pernambuco, Recife, PE, Brazil.

**Keywords:** Nutritional status, Bone density, Adults

## Abstract

**Objective:**

To investigate the relationship between total and segmental body fat, bone mineral density and bone mineral content in undergraduate students stratified according to nutritional status.

**Methods:**

The study included 45 male undergraduate students aged between 20 and 30 years. Total and segmental body composition, bone mineral density and bone mineral content assessments were performed using dual energy X-ray absorptiometry. Subjects were allocated into three groups (eutrophic, overweight and obese).

**Results:**

With the exception of upper limb bone mineral content, significantly higher (p<0.05) mean bone mineral density, bone mineral content, and relative body fat values were documented in the obese group. Total body and segmental relative body fat (lower limbs and trunk) were positively correlated (p<0.05) with bone mineral density in the overweight group. Upper limb fat was negatively correlated (p<0.05) with bone mineral content in the normal and eutrophic groups.

**Conclusion:**

Total body and segmental body fat were correlated with bone mineral density and bone mineral content in male undergraduate students, particularly in overweight individuals.

## INTRODUCTION

Bone mineral density (BMD) reflects a dynamic process that involves bone formation and resorption, and is described as bone remodeling. Bone mass is expressed as bone mineral content (BMC), g or kg and BMD (g/cm^2^), both influenced by bone size.

Maintenance of BMD is a key factor in osteoporosis prevention. In osteoporosis, bone matrix and mineral loss occur in response to excessive bone resorption relative to formation, with marked decrease in BMD.^(1,2)^ Adequate bone mineralization depends upon three potentially interrelated factors: circulating levels of hormones acting on the calcification process, mechanical overload of the skeleton and intake of sufficient amounts of calcium and vitamin D, along with endogenous vitamin D production.^(2,3)^

The effects of body composition (body fat, lean body mass and respective indices) on BMD have been investigated.^(4,5)^ According to Kang et al.,^(6)^ total body lean mass is a positive determinant of BMD, while the role of total body fat remains controversial. However, potential positive impacts of total body fat on BMD have been proposed, suggesting that fat may act as a substrate for conversion of androgens to estrogens,^(7)^ which are known to be associated with BMD.^(8,9)^

Still, deeper investigations of the relations between fat in the lower limbs, upper limbs and trunk, BMD and BMC are lacking, particularly in settings involving subjects of different levels of nutritional status, as is often the case. In contrast with the high BMD observed in overweight or obese individuals, low BMD values were reported in individuals with low body mass index (BMI); therefore, overweight individuals seem to enjoy better protection against osteoporosis and bone fractures compared to their eutrophic, low body weight peers.^(10,11)^

## OBJECTIVE

To investigate the relation between total and segmental body fat, bone mineral density and bone mineral content in young undergraduate students stratified according to nutritional status.

## METHODS

### Sample

This is a cross-sectional, descriptive, correlational study.^(12)^ The intentional, non-probabilistic sample comprised 45 male undergraduate students aged between 20 and 30 years, who volunteered to participate in the study and met the following inclusion criteria: young adult male; eutrophic, overweight or obese according to BMI classification.^(13)^ The exclusion criteria were as follows: transient or permanent physical condition precluding dual energy x-ray absorptiometry (DEXA), such as limb amputation, cardiac pacemaker or any other metallic implant, or use of kidney disease medication.

### Procedures

Participants were informed about study objectives and procedures. Anthropometric assessments were then scheduled at the *Laboratório de Avaliação da Performance Humana* (Human Performance Assessment Laboratory) and DEXA performed at *Laboratório Albuquerque do Ó* during standard working hours (10:00 am – 2:00 pm). This study was approved by the Ethics in Human Research Committee of *Centro de Ciências da Saúde da Universidade Federal de Pernambuco* (UFPE; registration no. 281/2004).

### Anthropometric and body composition assessments

Anthropometric and body composition assessments were performed according to the International Society for the Advancement of Kinanthropometry (ISAK) standards.^(14)^ Body mass was measured using a platform scale (Filizola^®^, Brazil; capacity and accuracy, 150kg/0.1kg), with subjects barefoot and wearing light clothing. Body height was measured to the nearest 0.1cm using a wooden stadiometer. Body mass index was defined as body mass divided by the square of body height [mass (kg)/height^2^(m)]. Subjects were allocated into three groups according to BMI, as follows: eutrophic (BMI 8.5 to 24.9kg/m^2^), overweight (BMI 25.0 to 29.9kg/m^2^) or obese (BMI above 30.0kg/m^2^).^(13)^

Body composition was assessed via densitometry (DEXA) using a total body pencil beam densitometer (Lunar DPX-L, Lunar Radiation, Madison, WI. USA) and 3.65 software. Radiation doses were below 1.0 mRem. The following variables were quantified by the same radiology technician: BMD, total and segmental body BMC (BMC_total_, BMC_upper_, BMC_lower_ and BMC_trunk_), as well as total and segmental body fat percentages (%F_total,_ %F_upper_, %F_lower_ and %F_trunk_).

### Statistical analysis

Numerical data were tested for normality using the Kolmogorov-Smirnov test. Exploratory data analysis was carried out to detect inconsistent data and outliers. Descriptive statistics parameters were expressed as mean and standard deviation (M±SD). Anthropometric and body composition variables (%F_total,_ %F_upper_, %F_lower_ and %F_trunk;_ BMD_total_, BMC_upper_, BMC_lower_ and BMC_trunk_) were compared via repeated measures analysis of variance (ANOVA) and the Bonferroni *post hoc* test. Correlations between total and segmental body composition, BMD and BMC were tested using the Pearson product–moment correlation coefficient. Statistical analyses were performed using software (Statistical Package for Social Science, SPSS – version 17.0 for windows). The level of significance was set at 5%.

## RESULTS

The mean values of each variable considered in the analysis are given per group in table 1. With the exception of BMC_upper_, values were higher in the obese compared to the eutrophic and overweight groups.

**Table 1:** Anthropometric indicators, body composition and bone mineral content according to nutritional status

Variables	Eutrophic n=16	Overweight n=15	Obese n=14
Body mass (kg)	70.0±5.1	83.1±5.9*	98.5±7.2*†
Height (cm)	175.9±5.8	174.6±.4	176.0±5.7
Body mass index (kg/m^2^)	22.6±1.5	27.2±1.0*	31.7±1.3*†
Relative body fat (%)	19.6±6.9	30.6±5.7*	35.4±4.6*
Relative upper limb fat (%)	15.6±7.3	33.1±7.6*	41.5±8.0*†
Relative lower limb fat (%)	20.3±6.7	28.5±5.9*	33.5±4.8*
Relative trunk fat (%)	20.0±7.1	31.3±5.2*	34.2±3.4*
Total body bone mineral density (g/cm^2^)	1.170±0.05	1.234±0.05*	1.253±0.52*
Upper limb bone mineral content (kg)	0.36±0.05	0.38±0.02	0.30±0.06*
Lower limb bone mineral content (kg)	1.11±0.12	1.21±0.12	1.29±0.14*
Trunk bone mineral content (kg)	0.96±0.11	1.02±0.10	1.08±0.10*

*Differs significantly from eutrophic; † Differs significantly from overweight; p<0.05. Mean ± standard deviation.

**Figure 1:**
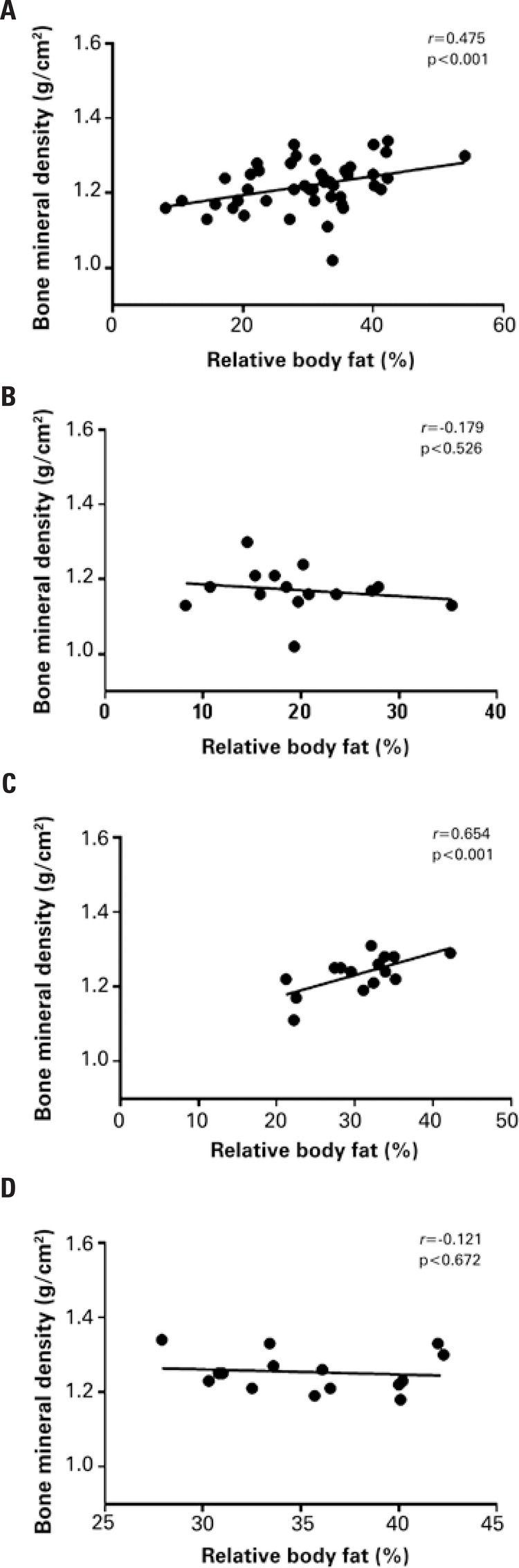
. Correlation between relative body fat and bone mineral density. (A) All subjects (global analysis); (B) Eutrophic group; (c) Overweight group; (D) Obese group

Investigation of potential correlations between body composition and BMD_total_ were based on separate, global and per group analyses. Global analysis revealed a significant positive correlation between BMD and body fat percentage; a similar correlation was observed in the overweight group (Figure 1).

Per group correlation analyses were carried out to investigate potential relations between nutritional status and body fat percentage, total body and segmental BMD, and total body and segmental BMC. Significant correlations were detected, they varied among groups. In the global analysis, total and segmental body fat percentages were positively correlated with BMD_total_ and BMC_lower_, while BMC_upper_ were negatively correlated with %F_lower_ and %F_upper_. In the eutrophic group, %F_upper_ was negatively correlated with BMC_upper_. In the overweight group, BMD_total_ was positively correlated with total and segmental body fat percentages. In the obese group, negative correlations between BMC_upper_, total and segmental body composition were documented (Table 2).

**Table 2:** Correlation (Pearson correlation coefficient, *r*) between fat percentage, bone mineral density and bone mineral content

	All subjects (global analysis)				Eutrophic group				Overweight group				Obese group			
			
	BMD_total_	BMC_trunk_	BMC_lower_	BMC_upper_	BMD_total_	BMC_trunk_	BMC_lower_	BMC_upper_	BMD_total_	BMC_trunk_	BMC_lower_	BMC_upper_	BMD_total_	BMC_trunk_	BMC_lower_	BMC_upper_
%F_total_	0.512*	0.284	0.359†	-0.477	-0.212	-0.331	-0.262	-0.401	0.680*	0.083	0.188	-0.227	-0.258	-0.187	0.123	-0.892*
%F_lower_	0.438*	0.289	0.378*	-0.544*	-0.138	-0.261	-0.232	-0.499	0.632†	0.299	0.190	-0.008	-0.187	-0.039	0.222	-0.731*
%F_upper_	0.512*	0.266	0.384*	-0.585*	-0.288	-0.460	-0.427	-0.599†	0.603†	-0.003	-0.066	-0.031	0.060	-0.035	0.202	-0.869*

*Significant correlation <0.001; † significant correlation <0.05. %F_trunk_: trunk fat percentage; %F_lower_: lower limb fat percentage; %F_upper_: upper limb fat percentage.

## DISCUSSION

This study set out to investigate the relations between total and segmental body composition, total and segmental BMD and total and segmental BMC in young undergraduate students stratified according to BMI. Body fat percentage was positively correlated with BMD in the global analysis (all individuals in the sample) and in the overweight group. However, intragroup per segment analyses revealed significant correlations between specific body segments, which differed in degree and magnitude according to BMI.

Several factors are thought to influence BMD and BMC, and body weight seems to be an important determinant of BMD.^(15)^ Analyses in this study were not focused on body weight; still the contribution of body weight is implicit, since body composition changes to the detriment of body weight variations. Thus, anthropometric parameters, total and segmental body composition, total and segmental BMD and total and segmental BMC gradually and significantly increased when groups were compared. However, obese individuals had the lowest BMC_upper_ values.

According to Dishman et al.,^(16)^ individuals with higher body mass indices also have higher BMC. In a study by Coin et al.^(17)^ involving elderly men and women with low to normal body mass, high risk of fractures and significant decrease in BMD leading to osteoporosis were reported in women with low body mass. The skeleton is highly adaptive to stimuli and, regardless of body composition (*i.e*., fat or lean muscle tissue), excess body weight translates into mechanical forces which act on bones and stimulate osteogenesis.^(18)^

The results of this study should be interpreted in light of the fact that body composition on a per segment basis was considered in the analysis, as opposed to fractional body composition (*e.g*., fat and fat-free mass). Also, the scarcity of studies involving male undergraduate students makes comparisons difficult and limits the possibility of making inferences, at least in part.

This study involved a first, global analysis of the relation between relative body fat and BMD. This introduced high heterogeneity of group and variables, increasing the spectrum of variability in the global analysis of the sample. Therefore, relations might have been influenced by the “group factor”, bearing in mind the different analyses of target components within groups. According to Raudenbush et al.,^(19)^ low coefficients of variation of values attributed to one or more variables may imply correlation coefficients close to zero when two variables are compared, affecting the direction of intervariable relations.

In a study involving 503 adult male subjects aged between 20 and 89 years and stratified according to different obesity criteria Kang et al.,^(6)^ relative fat and fat-free body mass were positively related with BMD at different body sites in all subjects. However, a significant negative relation was documented in the overweight group.

Body composition basically refers to two components - fat and lean body mass. Several studies investigated the association between body composition measures and BMD.^(20)^ However, the relative effect of each component on BMD remains to be determined. Lean body mass is known to be strongly related with BMD in men,^(20,21)^ but positive relations between fat body mass and BMD have also been reported.^(20,22)^

This study revealed correlations of different magnitudes between groups. Upper limb fat percentage (%F_upper_) was negatively correlated with BMC_upper_ in eutrophic subjects. In the overweight group, a positive relation was observed, but was limited to total and segmental body fat percentages and BMD. In the obese group, total and segmental body fat percentages were negatively correlated with BMC_upper._ These findings support the hypothesis that body composition and body fat distribution may play a role in the explanation of BMD and BMC relations.

Studies analyzing relations between body composition parameters, BMD and BMC are notably based on body fat percentage, total body fat and lean body mass, in an effort to identify the best predictor of such relations. However, to our understanding there are important aspects to be considered in this sort of analysis. Collinearity of variables, for instance, may result in the analysis of similar aspects due to superimposition of variables in the explanation of intervariable relations. This is not mentioned in studies found in the literature.

Age-related changes in body composition translate into increased body fat and decreased lean body mass in both, men and women. However, the different effects of age-related body composition changes on BMD and BMC in younger and older men remain to be determined.^(20)^ Also, it could be argued that, just as the presence and rate of bone mass loss vary widely across body sites, the relative contribution of body fat and lean body mass may be site-related.^(20,21)^

Ethnical, methodological (*e.g*., sampling sites) and age-group-related differences may explain the discrepancy of results between studies. In a study by El Hage et al.,^(23)^ involving 65 male and 35 female adolescents and investigating the relation between BMD and body composition, lean body mass was shown to be the best predictor of BMD. But positive effects of fat tissue on BMC and BMD in elderly individuals were also demonstrated.^(5)^ Yet, none of these findings shed light on the mechanism through which body composition components may affect BMD and BMC.

The cross-sectional nature of this study precludes the establishment of causal relations between selected variables. Bone mineral density and BMC values were compared between different subjects and thus may not represent true bone mass variability. Also, potential confounding variables, such as dietary habits, vitamin D levels and circulating levels of hormones that affect BMD and BMC have not been analyzed. Future studies on the relations between BMD, BMC and body composition in young undergraduate students including the aforementioned confounding variables are therefore warranted.

## CONCLUSION

Total and segmental body fat were related to bone mineral density and bone mineral content in the sample studied (young, male undergraduate students), particularly in overweight individuals. However, this relation appears to differ according to nutritional status, given the positive impacts of relative body fat on bone mineral density in overweight individuals.
