# Supplementary material for: Relation between body composition and bone mineral density in young undregraduate students with different nutritional status
Source: Einstein (Sao Paulo). 2016 Jan-Mar;14(1):12–7. doi: 10.1590/S1679-45082016AO3569 (PMC4872911; doi:10.1590/S1679-45082016AO3569)
Supplement: Translation

# Translation: Relação entre composição corporal e densidade mineral óssea em jovens universitários com diferentes estados nutricionais

**Authors:** Edil de Albuquerque Rodrigues, Marcos André Moura dos Santos, Amanda Tabosa Pereira da Silva, Breno Quintella Farah, Manoel da Cunha Costa, Florisbela de Arruda Camara e Siqueira Campos, Ana Patrícia Siqueira Tavares Falcão

**Affiliations:** 1 Universidade de Pernambuco, Recife, PE, Brasil.; 2 Universidade Federal de Pernambuco, Recife, PE, Brasil.

**Keywords:** Estado nutricional, Densidade óssea, Adultos

## Abstract

**Objetivo:**

Examinar a relação entre as gorduras corporal total e corporal segmentar com a densidade mineral óssea e conteúdo mineral ósseo em jovens universitários estratificados segundo o estado nutricional.

**Métodos:**

Participaram do estudo 45 estudantes homens entre 20 e 30 anos de idade. Foram realizadas avaliações da composição corporal, densidade mineral óssea e conteúdo mineral ósseo (total e segmentado) foram avaliados por meio da absortometria radiológica de dupla energia. Os sujeitos foram divididos em três grupos (eutrófico, sobrepeso e obesos).

**Resultados:**

Os obesos tiveram maiores valores médios nas variáveis de densidade mineral óssea, conteúdo mineral ósseo e gordura relativa comparativamente aos eutróficos e àqueles com sobrepeso (p<0,05 para todos), exceto no conteúdo mineral ósseo nos membros superiores. A gordura relativa total, bem como segmentar (membros inferiores e tronco), correlacionou-se positivamente com a densidade mineral óssea somente nos sobrepesados (p<0,05 para todos). Nos eutróficos e obesos, a gordura dos membros superiores foi correlacionada negativamente com o conteúdo mineral ósseo (p<0,05).

**Conclusão:**

Gordura corporal total e gordura corporal segmentada estiveram relaciondas com a densidade mineral óssea e o conteúdo mineral ósseo em jovens universitários masculinos, sobretudo em indivíduos com sobrepeso.

## INTRODUÇÃO

A densidade mineral óssea (DMO) é o resultado de um processo dinâmico de formação e reabsorção do tecido ósseo, conhecido como remodelação. A massa óssea é relatada em termos de conteúdo mineral ósseo (CMO), em gramas ou quilogramas, e a DMO, em g/cm^2^, sendo que ambos podem ser influenciados pelo tamanho do osso.

A manutenção da DMO é muito importante para a prevenção da osteoporose, que se caracteriza por uma diminuição acentuada da DMO, na qual a matriz e os minerais ósseos são perdidos devido ao excesso de reabsorção óssea em relação à formação.^1,2^ Para que ocorra uma adequação da mineralização óssea, três prováveis fatores devem se inter-relacionar: os níveis de hormônios circulantes que agem no processo de calcificação, a sobrecarga mecânica imposta ao esqueleto, além da ingestão adequada de cálcio e vitamina D, e sua produção.^2,3^

No entanto, alguns estudos têm explorado e discutido o efeito dos parâmetros da composição corporal (massa gorda, índice de massa gorda, massa magra e o índice de massa magra) sobre a DMO.^4,5^ Para Kang et al.,^6^ a massa magra total é um determinante positivo para o aumento da DMO, enquanto que o papel da massa gorda total ainda é controverso. No entanto, estudos indicam que a massa gorda total também atua positivamente sobre a DMO, sugerindo que a gordura pode ser substrato para conversão de andrógenos para estrógenos,^7^ que estão relacionados com a DMO.^8,9^

Todavia, a relação entre a gordura localizada nos membros inferiores, nos superiores e no tronco com a DMO e o CMO tem sido superficialmente investigada, sobretudo nos diferentes níveis de estado nutricional, que, de alguma forma, reflete uma exposição às condições oferecidas pelo ambiente atual. Neste cenário, tem sido descrito que indivíduos com baixo índice de massa corporal (IMC) apresentam menores valores de DMO, enquanto que indivíduos com sobrepeso e obesos apresentam níveis elevados de DMO, demonstrando que os indivíduos acima do peso têm maior proteção em relação a osteoporose e fraturas comparados aos seus pares eutróficos ou com baixo peso.^10,11^

## OBJETIVO

Examinar a relação entre as gorduras corporal total e corporal segmentar com a densidade mineral óssea e conteúdo mineral ósseo em jovens universitários estratificados segundo o estado nutricional.

## MÉTODOS

### Amostra

Este estudo caracteriza-se como transversal descritivo e correlacional.^12^ A amostra não probabilística e intencional foi composta por 45 estudantes universitários homens, com idades entre 20 e 30 anos, que aceitaram participar de forma voluntária do estudo, obedecendo aos critérios de inclusão estabelecidos: sexo masculino, adulto jovem, e estar dentro das categorias do IMC consideradas como eutrófico, sobrepeso e obeso.^13^ Foram critérios de exclusão: indivíduos que apresentaram algum problema de ordem física, temporário ou permanente; e que impedissem a realização da absortometria radiológica de dupla energia (DEXA); como amputação, ser portador de marca-passo ou qualquer metal no corpo, além da utilização de medicamentos renais.

### Procedimentos

Inicialmente, os indivíduos foram devidamente esclarecidos sobre os objetivos e os procedimentos adotados na pesquisa. Em seguida, foram agendados horários no Laboratório de Avaliação da *Performance* Humana, onde foram realizadas as avaliações antropométricas. Os exames DEXA foram realizados no Laboratório Albuquerque do Ó, em horário padrão, entre 10 e 14h. Este estudo foi aprovado pelo Comitê de Ética em Pesquisa envolvendo seres humanos do Centro de Ciências da Saúde da Universidade Federal de Pernambuco (UFPE), com o registro número 281/2004.

### Antropometria e composição corporal

Todas as avaliações antropométricas e da composição corporal foram tomadas utilizando técnicas convencionais descritas pela *International Society for the Advancement of Kinanthropometry* (ISAK).^14^ A massa corporal foi medida em uma balança de plataforma (Filizola^®^, Brasil), com carga máxima de 150kg e precisão de 0,1kg. Os sujeitos deveriam estar descalços e usando um menor número de roupas. Para a medida de estatura, foi utilizado um estadiômetro de madeira fixado à parede com precisão de 0,1cm. O IMC foi calculado dividindo-se a massa corporal pela estatura ao quadrado [massa (kg)/altura^2^(m)]. Com base nos valores encontrados no IMC, os indivíduos foram subdivididos em três grupos distintos. O primeiro grupo foi caracterizado como eutrófico (IMC entre 18,5 e 24,9kg/m^2^), sobrepeso (IMC entre 25,0 e 29,9kg/m^2^) e obeso (IMC acima de 30,0kg/m^2^).^13^

Para a avaliação da composição corporal, foi utilizado o método de densitometria corporal. A técnica empregada foi a DEXA, com equipamento modelo *Boné Densitometer Pencil-beam DPX Lunar Radiation* (Madison, WI. USA), utilizando *software* 3.65. A dose de radiação recebida é menor do que 1,0 mRem. Todos os exames foram realizados pelo mesmo técnico em radiologia. Dessa forma foram quantificadas as variáveis DMO, CMO total (CMO_total_) e segmentado (CMO_sup_, CMO_inf_ e CMO_tronco_), porcentual de gordura (%G_total_) e segmentar (%G_tronco_, %G_inf_ e %G_sup_).

### Análise estatística

Previamente ao início da análise, a normalidade dos dados numéricos foi analisada por meio dos testes de Kolmogorov-Smirnov. Uma análise exploratória foi realizada para identificar informações inconsistentes, como também a presença de *outliers*. Os dados da estatística descritiva estão apresentados em média e desvio padrão (M±DP). Para a comparação das variáveis antropométricas e da composição corporal (%G_total_, %G_sup_, %G_inf,_ %G_tronco_, DMO_total_, CMO_sup_, CMO_inf_ e CMO_troco_), utilizou-se Análise de Variância (ANOVA) para medidas repetidas, seguida de teste Bonferroni, como *post hoc*. O coeficiente de correlação momento-produto de Pearson foi adotado para análise da correlação entre a composição corporal, a DMO e o CMO, total e por segmentos. Em todas as análises, o nível de significância de 5% foi utilizado. Para a análise estatística, utilizou-se o *software Statistical Package for Social Science* (SPSS) para Windows, versão 17.0.

## RESULTADOS

A tabela 1 apresenta os valores médios encontrados em cada variável estratificados por grupo. Em todas as variáveis, exceto no CMO_sup_, os obesos foram os que apresentaram os maiores valores, comparativamente aos grupos eutrófico e com sobrepeso.

**Tabela 1:** Indicadores antropométricos, composição corporal e conteúdo mineral ósseo estratificado pelo estado nutricional

Variáveis	Eutróficos n=16	Sobrepeso n=15	Obesos n=14
Massa corporal (kg)	70,0±5,1	83,1±5,9*	98,5±7,2*†
Estatura (cm)	175,9±5,8	174,6±,4	176,0±5,7
Índice de massa corporal (kg/m^2^)	22,6±1,5	27,2±1,0*	31,7±1,3*†
Gordura relativa total (%)	19,6±6,9	30,6±5,7*	35,4±4,6*
Gordura relativa do braço (%)	15,6±7,3	33,1±7,6*	41,5±8,0*†
Gordura relativa da perna (%)	20,3±6,7	28,5±5,9*	33,5±4,8*
Gordura relativa do tronco (%)	20,0±7,1	31,3±5,2*	34,2±3,4*
Densidade mineral óssea total (g/cm^2^)	1,170±0,05	1,234±0,05*	1,253±0,52*
Conteúdo mineral ósseo superior (kg)	0,36±0,05	0,38±0,02	0,30±0,06*
Conteúdo mineral ósseo inferiror (kg)	1,11±0,12	1,21±0,12	1,29±0,14*
Conteúdo mineral ósseo tronco (kg)	0,96±0,11	1,02±0,10	1,08±0,10*

*Diferença significativa entre eutrófico; †diferença significativa entre sobrepeso; p<0,05. Média ± desvio padrão.

Para verificar a relação entre a composição corporal e a DMO_total_, foi realizada uma análise em toda amostra e em cada grupo. Em toda amostra, houve correlação significativa positiva entre a DMO e o porcentual de gordura. Essa correlação também foi observada no grupo sobrepeso (Figura 1).

**Figura 1:** Correlação entre a gordura relativa total e a densidade mineral óssea. (A) Todos os indivíduos; (B) eutróficos; (C) sobrepeso; (D) obesos

Com a finalidade de verificar a influência do estado nutricional em relação ao porcentual de gordura, a DMO e CMO total e por segmentos, foi realizada uma análise de correlação em cada grupo estudado. Os resultados demonstraram existência de correlações, mas com magnitudes diferenciadas entre os grupos. Em toda amostra, o %G_total_ e os porcentuais por segmentos apresentaram correlação significativa positiva com DMO_total_ e CMO_inf_; CMO_sup_ correlacionou-se negativamente com %Gord_inf_ e %Gord_sup_. Nos eutróficos, o %Gord_sup_ se correlacionou negativamente com CMO_sup_. No grupo sobrepeso, a DMO_total_ apresentou correlação significativa positiva com %Gord_total_ e segmentar. Já no grupo dos obesos, a correlação foi negativa e ocorreu entre o CMO_sup_ e a composição corporal total e por segmentos (Tabela 2).

**Tabela 2:** Correlação (correlação de Pearson - coeficiente *r*) entre o porcentual de gordura, a densidade mineral óssea e o o conteúdo mineral ósseo

	Todos				Eutrófico				Sobrepeso				Obeso			
	DMO_total_	CMO_tronco_	CMO_inf_	CMO_sup_	DMO_total_	CMO_tronco_	CMO_inf_	CMO_sup_	DMO_total_	CMO_tronco_	CMO_inf_	CMO_sup_	DMO_total_	CMO_tronco_	CMO_inf_	CMO_sup_
%G_total_	0,512*	0,284	0,359†	-0,477	-0,212	-0,331	-0,262	-0,401	0,680*	0,083	0,188	-0,227	-0,258	-0,187	0,123	-0,892*
%G_inf_	0,438*	0,289	0,378*	-0,544*	-0,138	-0,261	-0,232	-0,499	0,632†	0,299	0,190	-0,008	-0,187	-0,039	0,222	-0,731*
%G_sup_	0,512*	0,266	0,384*	-0,585*	-0,288	-0,460	-0,427	-0,599†	0,603†	-0,003	-0,066	-0,031	0,060	-0,035	0,202	-0,869*

*Correlação significante <0,001; †correlação significantes <0,05. %G_tronco_: porcentual de gordura do tronco; %G_inf_: porcentual de gordura de membros inferiores; %G_sup_: porcentual de gordura de membros superiores.

## DISCUSSÃO

O objetivo deste estudo foi examinar a relação entre a composição corporal total e segmentada, a DMO, e o CMO_total_ e por segmentos em jovens universitários estratificados de acordo com seu IMC. Os resultados demonstraram que o porcentual de gordura correlacionou-se positivamente com DMO em todos os sujeitos e no grupo sobrepeso. Porém, quando essas análises foram realizadas em cada grupo e por segmentos, foram identificadas correlações significativas, sendo que tais relações ocorrem em segmentos corporais específicos com grau e magnitudes diferenciados, segundo o IMC.

Muitos fatores parecem influenciar na DMO e CMO. No entanto, o peso corporal parece ser um determinante importante da DMO.^15^ No presente estudo, apesar de o peso corporal não ter sido o objeto central nas análises de correlação, sua participação está implícita às análises, uma vez que a composição corporal sofre alterações em detrimento das variações do peso corporal. Neste contexto, foi possível observar que os parâmetros antropométricos, da composição corporal total e por segmentos, a DMO e CMO total e regionais, aumentaram gradualmente e significativamente quando os grupos foram comparados. No entanto, os obesos foram os que apresentaram o menor CMO_sup_.

Neste contexto, Dishman et al.^16^ destacam que indivíduos que apresentam maiores valores em relação à massa corporal são os que também apresentam maior CMO. Em estudo realizado em homens e mulheres idosas com baixa massa corporal e massa corporal normal, Coin et al.^17^ constataram que as mulheres com baixa massa corporal apresentaram alto risco para fraturas, além de reduções significativas da DMO, levando à osteoporose. O esqueleto tem grande capacidade de adaptação aos estímulos, de modo que o excesso de peso representado tanto pela gordura corporal, como pelo tecido musculoesquelético, exerce força mecânica sobre os óssos, estimulando a osteogênese.^18^

Um aspecto que se destaca para os resultados do presente estudo refere-se ao fato de não ter sido utilizado um modelo de análise tendo por base os valores da composição corporal de forma fragmentada (por exemplo: massa gorda e massa isenta de gordura), mas a composição por segmentos. Outro aspecto é a existência de poucos estudos com jovens universitários masculinos, o que dificulta e limita, ao menos em parte, as comparações e as ilações que possam ser realizadas.

No presente estudo, inicialmente os sujeitos foram analisados conjuntamente, em relação à gordura corporal relativa e à DMO. Neste cenário, os resultados encontrados tornam o grupo e as variáveis estudadas muito heterogêneos e, desse modo, aumentam o espectro de variação na população, quando analisada conjuntamente. Assim, é possivel que haja uma influência do fator grupo sobre as relações, pois as análises realizadas em cada grupo ocorreram de forma distinta entre os componentes estudados. Neste sentido, Raudenbush et al.^19^ destacam que um baixo coeficiente de variação para a faixa de valores de uma ou mais variáveis pode determinar um coeficiente de correlação próximo de zero quando se relaciona uma variável com a outra, afetando a direção dessas relações.

Kang et al.,^6^ por exemplo, verificaram em homens adultos (n=503; 20 a 89 anos de idade), estratificados segundo diferentes critérios para obesidade, que a gordura relativa e a massa isenta de gordura estiveram positivamente relacionados com a DMO em diferentes locais do corpo em todos os sujeitos. Isso, porém, ocorreu de forma significativamente negativa no grupo com sobrepeso.

A composição corporal consiste basicamente em dois componentes − massa gorda e massa magra. Uma variedade de estudos anteriores examinou as associações entre as medidas da composição corporal e da DMO.^20^ No entanto, o efeito relativo de cada componente sobre a DMO permanece controverso. Embora a massa magra seja fortemente relacionada com a DMO em homens,^20,21^ também há relatos de que a massa de gordura está positivamente relacionada com a DMO.^20,22^

No presente estudo, foi verificado a existência de correlações, porém a magnitude não foi a mesma entre os grupos. Por exemplo, nos eutróficos, a relação ocorreu negativamente entre %Gord_sup_ e CMO_sup_. No grupo com sobrepeso, a relação foi positiva, mas ocorreu entre o %Gord_total_ e segmentar, apenas com a DMO. Já entre os obesos, o %Gord_total_ e segmentar, e o CMO_sup_ foram correlacionados negativamente. Esses achados suportam a ideia da influência da composição corporal e da distribuição da gordura na explicação das relações com a DMO e CMO.

Notadamente, estudos que analisam as respostas do parâmetros da composição corporal e DMO e CMO, utilizam o porcentual de gordura, a massa gorda e a massa magra, na tentativa de encontrar o melhor preditor dessas relações. No nosso entendimento, contudo, existe um aspecto que deve ser considerado quando essas relações são analisadas. Por exemplo, os estudos não reportam a existência de colinearidade entre as variáveis analisadas, o que pode representar uma superposição das variáveis, na explicação das relações uma com a outra e, desse modo, analisando aspectos similares.

No entanto, as mudanças que ocorrem na composição corporal com a idade refletem um aumento de massa gorda em ambos os sexos, enquanto que ocorre um um declínio na massa magra. Neste contexto, existe um questionamento sobre o quanto da alteração relacionada à idade na composição corporal afetaria a DMO e CMO de forma diferente em homens mais jovens e mais velhos.^20^ Além disso, o aparecimento e a taxa de perda de massa óssea em vários locais do esqueleto são bastante diferentes, sendo também razoável supor que a contribuição relativa da massa magra e gordura possa variar com locais do esqueleto.^20,21^

As possíveis razões para essas discrepâncias podem incluir diferenças de raça, locais ou pontos analisados em cada estudo e a idade dos sujeitos. El Hage et al.,^23^ em um estudo realizado com adolescentes (n=65 meninos e 35 meninas), relacionaram a DMO e os componentes da composição corporal. Foi observado que a massa magra foi o melhor preditor da DMO. No entanto, tem sido demonstrado um efeito positivo do tecido adiposo sobre o CMO e a DMO em idosos, sendo maior que a massa magra.^5^ Esses apectos, porém, não deixam claro de que forma os componentes da composição corporal podem afetar a DMO e CMO.

No entanto, destaca-se que este estudo apresenta limitações pelo delineamento transversal, o que não permite estabelecer uma relação de causalidade entre as variáveis analisadas, uma vez que os valores da DMO e CMO foram relacionados entre sujeitos diferentes e podem não representar a variabilidade real em relação a massa óssea. Ademais, possíveis variáveis de confusão, como hábitos alimentares, vitamina D e níveis circulantes de hormônios que afetam a DMO e CMO, não foram analisadas no presente estudo. Portanto, futuros estudos devem levar em consideração essas variáveis de confusão na relação entre DMO e CMO com a composição corporal em jovens universitários.

## CONCLUSÃO

Gordura corporal total e gordura corporal segmentada estiveram relaciondas com a densidade mineral óssea e o conteúdo mineral ósseo em jovens universitários masculinos. Essa relação, porém, parece ser diferente entre os variados estados nutricionais, posto que nos indivíduos com sobrepeso a gordura relativa teve impacto positivo na densidade mineral óssea.
